# Enriched environment and visual stimuli protect the retinal pigment epithelium and photoreceptors in a mouse model of non-exudative age-related macular degeneration

**DOI:** 10.1038/s41419-021-04412-1

**Published:** 2021-12-04

**Authors:** Hernán H. Dieguez, Juan S. Calanni, Horacio E. Romeo, Agustina Alaimo, María F. González Fleitas, Agustina Iaquinandi, Mónica S. Chianelli, María I. Keller Sarmiento, Pablo H. Sande, Ruth E. Rosenstein, Damián Dorfman

**Affiliations:** 1 Laboratory of Retinal Neurochemistry and Experimental Ophthalmology, Department of Human Biochemistry, School of Medicine/CEFyBO, University of Buenos Aires/CONICET, Buenos Aires, Argentina; 2 School of Engineering and Agrarian Sciences, Pontifical Catholic University of Argentina, BIOMED/UCA/CONICET, Buenos Aires, Argentina; 3 Interdisciplinary Laboratory of Cellular Dynamics and Nanotools, Department of Biological Chemistry, School of Exact and Natural Sciences/IQUIBICEN, University of Buenos Aires/CONICET, Buenos Aires, Argentina

**Keywords:** Neurodegeneration, Retina

## Abstract

Non-exudative age-related macular degeneration (NE-AMD), the main cause of blindness in people above 50 years old, lacks effective treatments at the moment. We have developed a new NE-AMD model through unilateral superior cervical ganglionectomy (SCGx), which elicits the disease main features in C57Bl/6J mice. The involvement of oxidative stress in the damage induced by NE-AMD to the retinal pigment epithelium (RPE) and outer retina has been strongly supported by evidence. We analysed the effect of enriched environment (EE) and visual stimulation (VS) in the RPE/outer retina damage within experimental NE-AMD. Exposure to EE starting 48 h post-SCGx, which had no effect on the choriocapillaris ubiquitous thickness increase, protected visual functions, prevented the thickness increase of the Bruch’s membrane, and the loss of the melanin of the RPE, number of melanosomes, and retinoid isomerohydrolase (RPE65) immunoreactivity, as well as the ultrastructural damage of the RPE and photoreceptors, exclusively circumscribed to the central temporal (but not nasal) region, induced by experimental NE-AMD. EE also prevented the increase in outer retina/RPE oxidative stress markers and decrease in mitochondrial mass at 6 weeks post-SCGx. Moreover, EE increased RPE and retinal brain-derived neurotrophic factor (BDNF) levels, particularly in Müller cells. When EE exposure was delayed (dEE), starting at 4 weeks post-SCGx, it restored visual functions, reversed the RPE melanin content and RPE65-immunoreactivity decrease. Exposing animals to VS protected visual functions and prevented the decrease in RPE melanin content and RPE65 immunoreactivity. These findings suggest that EE housing and VS could become an NE-AMD promising therapeutic strategy.

## Introduction

Age-related macular degeneration (AMD) is the leading cause of irreversible blindness in developed countries, with global prevalence of ∼9% [1–3] probably increasing as population grows and ages [4]. There are two types of AMD: exudative and non-exudative (NE). NE-AMD accounts for ∼80% of the disease [4], known as geographic atrophy (GA), due to photoreceptor (PR) and retinal pigment epithelium (RPE) exclusively macular damage, leaving the peripheral retina undamaged. The RPE, widely accepted as the anatomic location where NE-AMD initiates (reviewed by Kaarniranta et al. [5]), is a monolayer of cells containing melanin lying upon the choroid and baring functions such as PR maintenance, high-energy photons and oxidative stress scavenging, and outer retina metabolic supporting, among others (reviewed by Sparrow et al. [6]). The RPE aerobic metabolic rate accounts for a high mitochondria mass, a major source of local reactive oxygen species (ROS) [1], and RPE oxidative stress and mitochondria damage is an early event within NE-AMD [3, 7–12].

We have recently developed a new model of NE-AMD induced by unilateral superior cervical ganglionectomy (SCGx) in C57Bl/6J mice, which reproduces the central hallmarks of the human disease. At 10 weeks post-SCGx, visual dysfunction, and PR outer segment (OS), Bruch’s membrane (BrM), RPE ultrastructural alterations, and RPE and PR apoptosis are observed exclusively at the central temporal retina [13], leaving the nasal and peripheral retina undamaged. Moreover, we have shown that at early stages (i.e., 6 weeks post-surgery), SCGx induces oxidative stress, and a decrease in RPE antioxidant enzymes and mitochondria mass, also at the central temporal region [14].

Despite new therapeutic interventions are being developed, available strategies are still unable to prevent or even delay the NE-AMD induced atrophy of the RPE and the consequent vision loss [15]. Enriched environment (EE) refers to a scenario that boosts sensory, cognitive, motor and social stimuli relative to standard conditions (standard environment (SE)). EE induces morphological and neurochemical changes, such as cortex thickening, dendritic spine density increase, neurotrophic factors expression and neurogenesis enhancement [16, 17]. Moreover, several studies have shown both structural and functional neuroprotective effects triggered by EE after stroke [18–23]. The retina is responsive to EE during development and early post-natal stages: EE accelerates the maturation of retinal acuity [24], enhances visual acuity in animals exposed from birth [25] and protects the retina against glutamate damage in neonatal rats [26]. More recently, it has been shown that the adult retina is also susceptible to EE modulation; EE induces neuroprotection against retinal ischaemia, diabetic retinopathy [27–29] and experimental glaucoma [30] in adult rats, and extends PR survival and visual function in a mouse model of retinitis pigmentosa [31, 32].

In this context, the aim of the present report was analysing the effects of environmental enrichment and visual stimulation (VS) on NE-AMD induced by SCGx in adult C57Bl/6J mice.

## Results

**Fig. 1:**
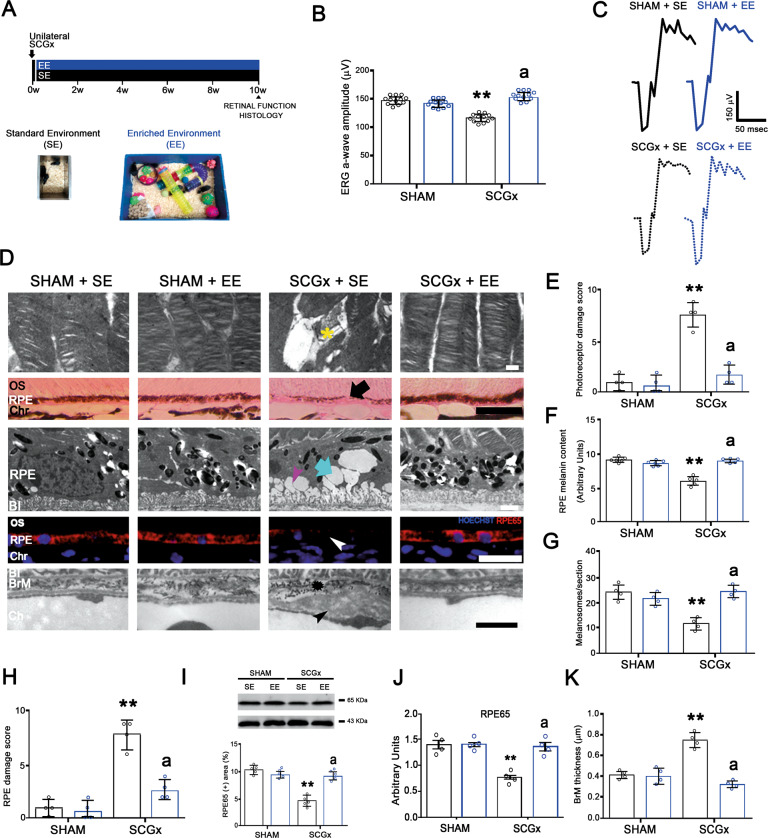
Effect of EE on the retinal dysfunction and histological alterations at 10 weeks post-SCGx. **A** Experimental protocols. **B** SCGx induced a significant decrease in ERG a-wave amplitude in animals housed in SE, whereas EE starting 48 h post-SCGx, which had no effect per se, completely prevented the decrease in this parameter (representative traces shown in **C**). Data are mean ± SEM (*n*: 12 animals per group), **P < 0.01 vs. sham-treated eyes from SE animals; a: *P* < 0.01 vs. SCGx-treated eyes from SE animals, by Tukey’s test. **D** In animals housed in SE, SCGx induced focal losses of PR discs (yellow asterisk) and blebs (quantified in **E**), and a decrease in RPE melanin content (black arrow) and melanosome number at the central temporal RPE, which were completely prevented by EE (quantified in **F** and **G**). EE also prevented RPE vacuolization (cyan arrow) and basal infolding thickening (magenta arrowhead) (quantified as RPE damage score in **H**), as well as the decrease in RPE65-immunostaining (white arrowhead) and protein levels at the temporal RPE at 10 weeks post-SCGx (quantified in **I** and **J**). SCGx induced BrM thickening (quantified in **K**), a clear loss of its pentalaminar structure (asterisk) and thickening of the endothelial cell basal membrane (black arrowhead), which were prevented by EE. Shown are representative photomicrographs from 5 (for optic microscopy) and 4 (for electronic microscopy) eyes/group, at 800 μm temporally from the ONH. OS photoreceptor outer segments, RPE retinal pigment epithelium, BI RPE basal infoldings, BrM Bruch’s membrane, Ch choriocapillaris, Chr choroid. Scale bars = 500, 20, 25, 1.5, 300 nm. Data are mean ± SEM (*n*: 5 (for optic microscopy) and 4 (for electronic microscopy) eyes per group), **P < 0.01 vs. sham-treated eyes from SE animals; a: *P* < 0.01 vs. SCGx-treated eyes from SE animals, by Tukey’s test. Data are mean ± SEM (*n*: 5 homogenates per group), **P < 0.01 vs. sham-treated eyes from SE animals, by Tukey’s test.

**Fig. 2:**
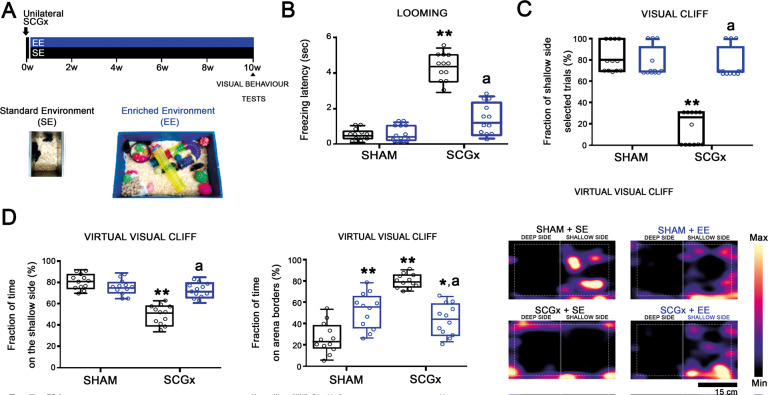
Effect of EE on the visual behaviour tests at 10 weeks post-SCGx. **A** Experimental protocols. **B**–**D** In SE animals, SCGx induced a significant increase in the freezing latency of the looming test, and a significant decrease both in the fraction of shallow-side selected trials in the visual cliff test, and the percentage of time spent on the shallow side in the virtual visual cliff test, which were prevented by EE. The time spent at the borders of the virtual visual cliff arena significantly increased in sham-treated animals housed in EE and SGCx-treated animals housed in SE and EE. Heatmaps of the tracked position of sham- and SCGx-treated eyes from SE and EE animals are shown. **P < 0.01 vs. SE animals with sham-treated eyes; a: *P* < 0.01 vs. SE animals with SCGx-treated eyes, by Tukey’s test (*n*: 12 animals per group).

**Fig. 3:**
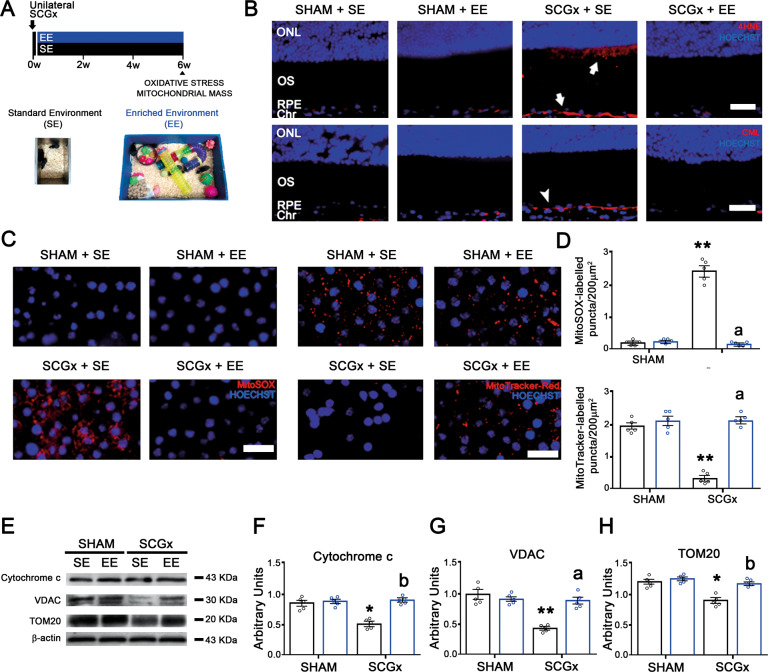
Effect of EE on the temporal outer retina/RPE oxidative damage, and RPE mitochondria mass at 6 weeks post-SCGx. **A** Experimental protocols. **B** EE prevented the SGCx-induced increase in 4HNE- and CML-immunoreactivity at the outer retina/RPE (arrow), and RPE (arrowhead), respectively. Shown are representative photomicrographs from 5 eyes/group, at 800 μm temporally from the ONH. ONL outer nuclear layer, OS photoreceptor outer segments, RPE retinal pigment epithelium, Chr choroid. Scale bars: 30 μm. **C** EE significantly prevented both the SCGx-induced increase in MitoSox-Red-labelled mitochondria and decrease in Mitotracker-Red(+) puncta at the RPE (quantified in **D**). Shown are representative photomicrographs from five eyes/group, at 800 μm temporally from the ONH. Scale bars = 20 μm. Data are mean ± SEM (*n*: 5 eyes per group), **P < 0.01 vs. sham-treated eyes from SE animals; a: *P* < 0.01 vs. SCGx-treated eyes from SE animals, by Tukey’s test. **E**–**H** SCGx induced a decrease in the levels of cytochrome *c*, VDAC, and TOM20 at the temporal RPE, which were prevented by EE. Data are mean ± SEM (*n*: 5 homogenates per group), **P < 0.01, *P < 0.05 vs. sham-treated eyes from SE animals; a: *P* < 0.01, b: *P* < 0.05 vs. SCGx-treated eyes from SE animals, by Tukey’s test.

**Fig. 4:**
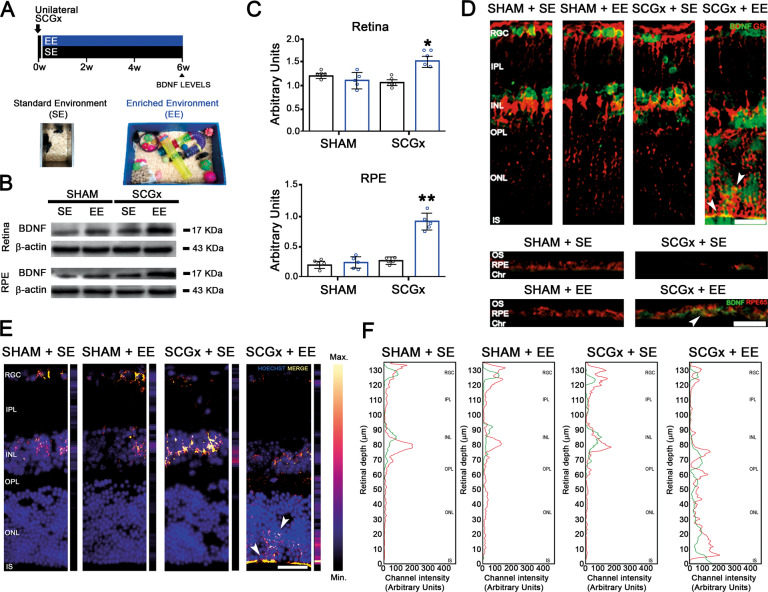
Effect of EE on the temporal retina/RPE BDNF-immunoreactivity and protein levels, and temporal retina BDNF/GS co-localization at 6 weeks post-SGCx. **A** Experimental protocols. **B**, **C** BDNF levels were significantly increased in SCGx-treated eyes from animals housed in EE both at the retina and RPE. Data are mean ± SEM (*n*: 5 homogenates per group), **P < 0.01, *P < 0.05 vs. sham-treated eyes from SE animals, by Tukey’s test. **D** EE, which had no effect on BDNF-immunoreactivity in sham-treated eyes, increased this parameter at the retina and RPE from SCGx eyes, particularly at the OPL, ONL and IS layers. Shown are representative photomicrographs at 800 μm temporally from the ONH from 5 eyes/group. RGC retinal ganglion cell layer, IPL inner plexiform layer, INL inner nuclear layer, OPL outer plexiform layer, ONL outer nuclear layer, IS PR inner segments, OS PR outer segments, RPE retinal pigment epithelium, Chr choroid. Scale bars = 30 μm. **E** Exclusive merge (yellow) from GS- and BDNF-immunoreactivity pictures were analysed using Python software. BDNF immunoreactivity was exclusively increased at the outer retina in SCGx-treated animals housed in EE. **F** Analysis of the red (GS) and green (BDNF) pixels from GS- and BDNF-immunoreactivity pictures also analysed using Python software. At the retinas from sham-treated eyes and SCGx-treated eyes from SE animals merge (**E**) and coincident peaks in red and green pixels (**F**) were evident mainly at the GCL and the INL, whereas in SCGx-treated eyes from EE animals, GS- and BDNF-immunoreactivity merge (**E**), and red and green pixel peaks (**F**) particularly increased at the outer retina and Müller cell end feet. Shown are representative photomicrographs from 5 eyes/group, at 800 μm temporally from the ONH. RGC retinal ganglion cell layer, IPL inner plexiform layer, INL inner nuclear layer, OPL outer plexiform layer, ONL outer nuclear layer, IS PR inner segments, OS PR outer segments, RPE retinal pigment epithelium, Chr choroid. Scale bars = 30 μm.

**Fig. 5:**
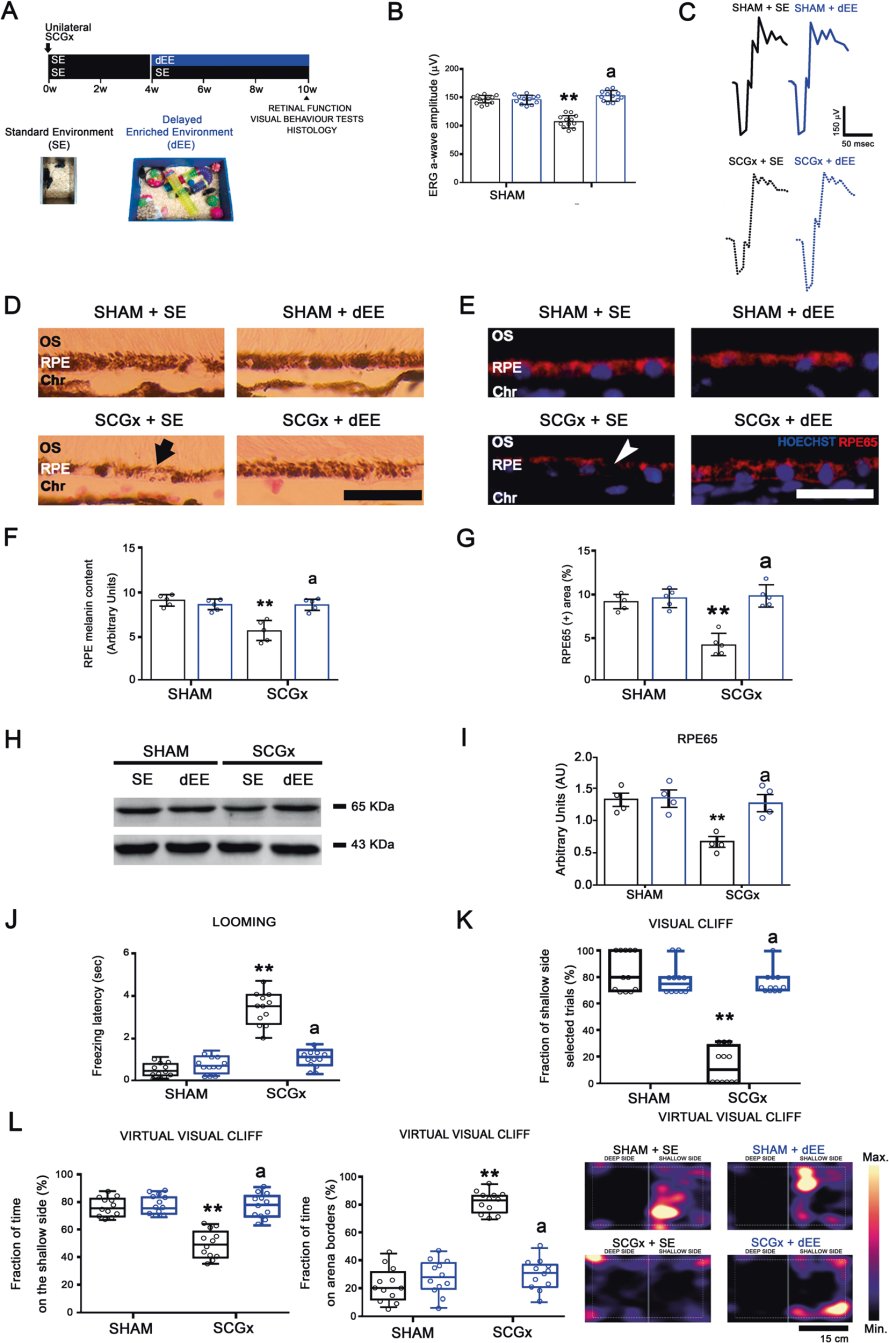
Effect of the delayed EE exposure (dEE) on the retinal function and temporal RPE structural alterations and visual behaviour tests at 10 weeks post-SCGx. **A** Experimental protocols. **B** EE starting at 4 weeks post-SCGx completely reversed the decrease in the ERG a-wave amplitude (representative traces shown in **C**). Data are mean ± SEM (*n*: 12 animals per group), **P < 0.01 vs. SE animals with sham-treated eyes; a: *P* < 0.01 vs. SE animals with SCGx-treated eyes, by Tukey’s test. **D**–**I** SCGx induced a decrease in the melanin content (arrow) and RPE65-immunoreactivity (arrowhead) and protein levels at the temporal RPE in SE animals. The delayed exposure to EE totally reversed these alterations. Shown are representative photomicrographs from 5 eyes/group at 800 μm temporally from the ONH. OS PR outer segments, RPE retinal pigment epithelium, Chr choroid. Scale bars = 25 μm. Data are mean ± SEM (*n*: 5 eyes per group), **P < 0.01 vs. sham-treated eyes from SE animals; a: *P* < 0.01 vs. SCGx-treated eyes from SE animals, by Tukey’s test. Data are mean ± SEM (*n*: 5 homogenates per group), **P < 0.01 vs. sham-treated eyes from SE animals, by Tukey’s test. **J**–**L** The delayed exposure to EE reversed the worse performance in the looming, visual cliff and virtual visual cliff tests induced by SCGx in SE-housed animals. **P < 0.01 vs. SE animals with sham-treated eyes; a: *P* < 0.01 vs. SE animals with SCGx-treated eyes, by Tukey’s test (*n*: 12 animals per group).

**Fig. 6:**
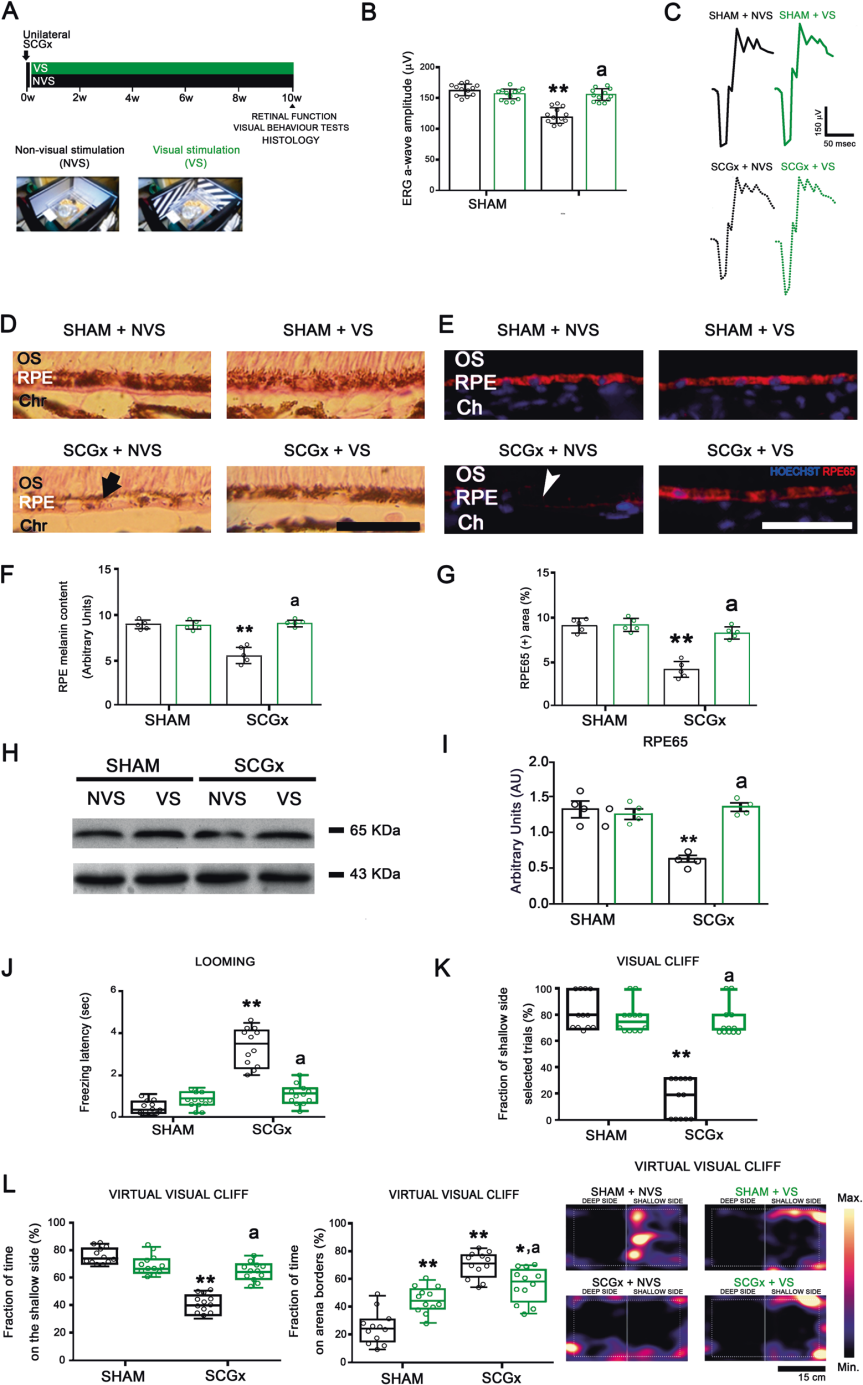
Effect of visual stimulation (VS) on the retinal function and temporal RPE structural alterations and visual behaviour tests at 10 weeks post-SCGx. **A** Experimental protocols. **B** SCGx induced a significant decrease in ERG a-wave amplitude in animals exposed to non-visual stimulation (NVS), whereas VS exposure since 48 h post-SCGx completely prevented the decrease in this parameter (representative traces shown in **C**). **D**–**I** Exposure to VS totally prevented the SCGx-induced decrease in the melanin content (arrow) and RPE65-immunoreactivity (arrowhead) and protein levels at the temporal RPE in SCGx-treated eyes from animals exposed to NVS. Shown are representative photomicrographs from 5 eyes/group, at 800 μm temporally from the ONH. OS photoreceptor outer segments, RPE retinal pigment epithelium, Chr choroid. Scale bars = 25 μm. Data are mean ± SEM (*n*: 5 eyes per group), **P < 0.01 vs. sham-treated eyes from animals exposed to NVS; a: *P* < 0.01 vs. SCGx-treated eyes from animals exposed to NVS, by Tukey’s test. Data are mean ± SEM (*n*: 5 homogenates per group), **P < 0.01 vs. sham-treated eyes from animals exposed to NVS, by Tukey’s test. **J**–**L** VS completely preserved the performance in the looming test, the visual cliff, and the virtual visual cliff tests. The time spent at the borders of the virtual visual cliff arena significantly increased in sham-treated animals housed in VS and SGCx-treated animals housed in NVS and VS. **P < 0.01 vs. sham-treated eyes from animals exposed to NVS; a: *P* < 0.01 vs. SCGx-treated eyes from animals exposed to NVS, by Tukey’s test (*n*: 12 animals per group).

Figure 1 summarizes the effect of EE-housing against the functional and structural alterations observed in the retina at 10 weeks post-SCGx. Although EE housing did not protect the structural changes induced by SCGx at the nasal and temporal choriocapillaris (Supplementary Fig. 1), it significantly prevented the decrease in the ERG a-wave amplitude induced by SCGx (Fig. 1B, C). We did not find differences in ERG b-wave amplitude, and ERG a- and b-wave latencies between experimental groups (Supplementary Table 1). At structural level, EE preserved OS integrity, RPE melanin content, RPE65-immunoreactivity, and protein levels, RPE melanosome number and ultrastructure, and BrM ultrastructure and thickness (Fig. 1D–K). SCGx did not induce alterations at the nasal outer retina in animals housed in SE, and EE had no effects both in sham-treated and SCGx-eyes at the nasal outer retina (Supplementary Fig. 2). The effect of EE on the alterations in vision-guided behavioural tests at 10 weeks post-SCGx is shown in Fig. 2. SCGx induced an increase in the freezing latency in the looming test (Fig. 2B), a decrease in the number of animals choosing the shallow side in the visual cliff test (Fig. 2C), and a decrease in the time spent at the shallow side of the virtual visual cliff (Fig. 2D), which were all prevented by EE. The effect of EE on oxidative damage at the temporal region was assessed at 6 weeks post-surgery (Fig. 3) to study the mechanisms by which EE could induce protection against NE-AMD. In animals housed in SE, SCGx induced an increase in OS and RPE oxidative stress markers 4-hydroxy-2-nonenal (4HNE) and carboxymethyl-lysine (CML), which was prevented by EE housing (Fig. 3B). Moreover, EE significantly prevented the increase in MitoSOX-immunoreactivity and the decrease in MitoTracker-Red-immunoreactivity at the temporal RPE (Fig. 3C, D). The decrease in RPE intrinsic mitochondria proteins (cytochrome *c*, voltage-dependent selective anion channel (VDAC) and translocase of the outer membrane (TOM20)) levels was significantly prevented by EE housing (Fig. 3E–H). To study the involvement of BDNF in EE-induced retinal protection, we assessed BDNF-immunoreactivity and its protein levels at the temporal retina and RPE at 6 weeks post-SCGx, as shown in Fig. 4. Even though EE had no effect in retinal and RPE BDNF levels and immunoreactivity in sham-treated eyes, it significantly increased these parameters at the temporal retina and RPE in SCGx-eyes (Fig. 4B, C). At the retina, BDNF-immunoreactivity increased particularly at the outer plexiform layer, the outer nuclear layer, and PR inner segments, and merged with glutamine synthetase (a Müller cell marker) (Fig. 4D), showed in detail in Fig. 4E, F. We found no differences in oxidative stress parameters, mitochondria protein levels and BDNF-immunoreactivity and levels at the nasal retina/RPE in sham- or SCGx-submitted eyes from animals in SE or EE (Supplementary Figs. 3 and 4). To study whether EE could not only prevent but also reverse the damage induced by SCGx, animals submitted to unilateral SCGx and housed in SE for 4 weeks post-SCGx were then segregated in SE or EE (i.e., delayed EE (dEE)) until 10 weeks post-surgery (Fig. 5A). The functional and histological alterations in the retinas from animals housed in SE for 10 weeks post-SCGx was completely reversed in animals exposed to dEE (Fig. 5B–I). Moreover, the visual behaviour was also protected by dEE (Fig. 5J–L). There were no differences between sham- or SCGx-treated eyes from animals housed in SE or dEE at the nasal outer retina/RPE (Supplementary Fig. 5). To analyse whether visual stimulation (VS) mimics the protective effect of EE, unilaterally sham- and SCGx-submitted animals were exposed to 12 h VS (i.e., 100% white–black contrast patterns) or non-VS (NVS) (full screen at 50% grey) at the light period for 10 weeks (Fig. 6A). VS significantly prevented the functional and histological retinal alterations induced by SCGx (Fig. 6B–I) and protected visual behaviour (Fig. 6J–L). VS had no effects at the nasal RPE both in sham- or SCGx-submitted animals (Supplementary Fig. 6).

## Discussion

The results shown herein show that the exposure of animals to EE or VS achieved both functional and structural RPE and outer retina protection against experimental NE-AMD. EE preserved visual function, and BrM thickness and morphology, as well as RPE/PR ultrastructure. As shown by previous works, SCGx induces ubiquitous choroid alterations [9, 13, 14, 33, 34]; however, with the exception of the choriocapillaris, all alterations induced by experimental NE-AMD induced by SCGx were exclusively located at the RPE/outer retina central temporal (without the central nasal or peripheral affection) region, which according to Volland and co-workers [35] is an area in the C57BL/6J mice comparable to the human macula due to the human-like cone/rod ratio, the highest cone concentration, and RPE/BrM specialization. Therefore, up to date, SCGx-induced NE-AMD is the only experimental model that mimics the temporal retina circumscribed damage, characteristic of human NE-AMD. In fact, we have shown that the localized damage induced by SCGx is not a direct consequence of ubiquitous choroid denervation and vascular alterations, but due to biochemical and structural differences between the nasal and temporal region [13, 14]. Experimental NE-AMD induced by SCGx can be divided in two phases: an early phase (up to 6 weeks post-SCGx), characterized by PR dysfunction (ERG a-wave), BrM thickening, decreased RPE melanin content, and RPE65-immunoreactivity, subtle ultrastructural RPE and PR alterations, increase in oxidation markers (4HNE, CML, MitoSOX), and mitochondria mass decrease, followed by a late phase (at 10 weeks post-SCGx), at which severe RPE ultrastructural damage and PR loss become evident, including RPE and PR apoptosis [13]. In the present report, we chose to analyse NE-AMD outcomes at 10 weeks post-SCGx to maximize the damage in the RPE/outer retina function and histology and discard the possibility of a transient EE- or VS-induced protection.

The ERG a-wave is widely recognized as an index of PR activity. The decrease in the scotopic ERG a-wave amplitude induced by SCGx was completely prevented in animals housed in EE. In agreement, EE preserves the ERG in rats with retinal ischaemia [27], type-1 diabetes [28], and retinitis pigmentosa [31], further supporting that EE protects PR function. We found no differences in ERG b-wave amplitude or ERG a- and b-wave latencies, indicating that ERG a-wave duration was similar between experimental groups.

BrM, a strategically located functional cluster [36], is a key player in hydraulic homeostasis, metabolic waste disposal and nutrient transport between systemic circulation, and avascular outer retina. BrM thickening leads to RPE and PR damage [37, 38]. Although it remains elusive whether BrM alterations lead to RPE damage or vice versa, EE exposure preserved BrM either as a primary effector or as consequence of RPE protection.

Melanin concentrates in the choroid and the RPE and plays a central role in radiation excess and RPE metabolism derived ROS removal [39]. Therefore, a decrease in the RPE melanin content might lead to a less effective quenching of both ROS and high-energy photons, with a consequent increase in local oxidative stress. RPE65, an isomerohydrolase exclusively located in the RPE that produces 11-*cis*-retinol from all-*trans*-retinyl esters, is a key player in the visual cycle whose alterations lead to irreversible blindness [40]. EE prevented the SCGx-induced decrease in both RPE melanin content and RPE65-immunoractivity and protein levels at the central temporal region.

Although ERG recording is a means to specifically assess retinal function, it only provides reliable information on the electrical response of the retina to a light flash, while vision comprises a process that is much more complex and involves extra-retinal nuclei. Therefore, we assessed the effect of NE-AMD and EE on three vision-dependent behavioural tests, allowing us to evaluate the visual function from a wider perspective. While the looming response is a visual cue exclusively relying reflex [41] that evaluates the integrity of the retino-colicular pathway, both the visual and the virtual visual cliff tests rely on the innate tendency of animals to avoid deep places and evaluate binocular depth perception (real or illusionary, respectively), as a measure of retino-geniculo-cortical pathway functional integrity [42, 43]. SCGx induced alterations in the performance in the three tests in animals housed in SE, which was completely prevented by EE exposure.

No doubt, NE-AMD is a complex disease, which involves a wide range of pathological mechanisms. However, oxidative damage, particularly located at the central temporal region of the RPE, appear to have a pivotal role in its starting point and progression. A surplus of ROS derived from mitochondria due to mitochondria malfunction has been reckoned as an early event both in human and experimental NE-AMD [3, 14, 44, 45]. Moreover, mitochondrial DNA damage [46] in AMD patients, a decrease in mitochondrial mass, and ultrastructural alterations in mitochondria have been observed in AMD specimens [44]. As shown herein, at 6 weeks post-SCGx EE prevented lipid peroxidation and superoxide levels in mitochondria increase, as well as the decrease in mitochondria mass induced by SCGx [13], proving that the exposure to EE protected RPE/PR function and structure through its antioxidant activity. Consistently with our results, it has been shown that EE protects mice brain neurons by increasing antioxidant activity [47], and maintaining rat brain mitochondria integrity [48], mass and function [49] after stroke.

BDNF, a key neurotrophin in brain circuits development, neuronal and synapse maintenance, and neuronal protection and regeneration [50], plays a major role in EE-mediated retinal neuroprotection [28, 30]. Müller cells are a major source of retinal BDNF [51, 52], and central players in retinal homeostasis [53]. Moreover, it has been demonstrated that BDNF is also synthetized in the RPE and might have both autocrine and paracrine activity [54–58]. At 6 weeks post-SCGx, together with the antioxidant effect, EE induced an increase in BDNF levels both in the central temporal RPE and whole retina homogenates. Notably, BDNF immunoreactivity was particularly increased at the outer retina and Müller cell end feet, close to PR inner segments where it merged with glutamine synthetase. In this line, it has been reported that serum and aqueous humour BDNF levels are lower in patients with NE-AMD [59], that BDNF intravitreal injections protects PR against light damage [60, 61], and that increased BDNF expression delays PR death and protects retinal function in a mouse model of primary PR degeneration [62]. In addition, BDNF enhances RPE cell survival [63]. Furthermore, an increase in Müller cell BDNF located at the outer retina protects the RPE against sodium iodate injury [64], and overexpression of RPE BDNF protects PR in a rat model of PR degeneration [65].

One of the main issues clinicians and researchers face is the timing in which chronic NE-AMD patients should begin treatment, to avoid early too aggressive therapies or starting too late and risk irreversibly loosing healthy tissue [66]. Although EE was capable to prevent visual function deficit and retinal structure damage within NE-AMD, two concerns arise: (i) the fact that EE-housing started shortly after SCGx (i.e., 48 h after surgery) could limit the potential clinical translation of these results as should require a too early diagnosis and (ii) EE implies a complex array of stimuli, which makes it difficult when attempting to translate it to humans. To analyse whether EE could not only prevent, but also slow or reverse NE-AMD ongoing progression, another set of animals were exposed to EE starting at 4 weeks post-SCGx, a time-point at which functional and structural alterations are already evident and might be comparable to the human early stage (and already diagnosed) disease [13]. The delayed exposure to EE reversed functional damage and achieved a complete protection of RPE melanin content and RPE65-immunoreactivity and protein levels, thus being capable of actively supressing ongoing NE-AMD damage.

Regarding the complexity of EE, evidence suggests that its brain effects imply the synergic effects of a wide range of stimuli [67, 68] adding an obstacle when attempting the application of EE paradigms to the clinical practise. Therefore, identifying the role of the individual EE components (e.g., social, sensory, motor) in achieving the protection elicited by the entire enriched experience is central when designing therapeutic strategies. In this context, recent evidence shows that VS, but not the other EE components protects the retina against acute ischaemia in rats [69]. As shown herein, the exposure to VS mimicked the functional and structural recovery induced by EE against NE-AMD, which could allow us to envision different strategies of VS (such as visual training or home-based videogames) that might benefit AMD patients, as previously shown for adult amblyopia recovery [70]. Although a cortical compensatory effect cannot be formally ruled out, the retinal function assessment (i.e., ERG) and structural analysis, together with the outcomes in the tests assessing visual behaviour, strongly support the retina as an anatomical locus for the protection induced by EE, dEE, and VS against NE-AMD. Therefore, despite the well-known differences between the mouse and human retina, the present results might be the beginning of a new era where pharmacotherapy is accompanied with potentially translatable non-invasive strategies that enhance mechanisms of protection, aimed to preserve visual capacity (even when the disease had already started), and avoid the dreaded visual dysfunction induced by NE-AMD.

## Materials and methods

### Animals and ethics

Adult male C57BL/6J mice (average weight of 27 ± 3 g and average age of 2.5 ± 0.5 months) were housed under controlled temperature, luminosity, and humidity, and under a 12-h light/12-h dark lighting schedule. The ethics committee of the School of Medicine, University of Buenos Aires (Institutional Committee for the Care and Use of Laboratory Animals (CICUAL) approved this study. All experiments conformed the guidelines on the care and use of animals adopted by the Society for Neuroscience and the Association for Research in Vision and Ophthalmology. For all experimental procedures, animals were anesthetized with intramuscular injection of 100 mg/kg ketamine hydrochloride and 2 mg/kg xylazine hydrochloride.

### Superior cervical ganglionectomy

The left superior cervical ganglion (SCG) was removed aseptically, as previously described [13], while a sham procedure was performed, without removing the right SCG, further considering the left eye as ganglionectomized (SCGx) and the right eye as control (sham).

### Enriched environment

Two days after SCGx, animals were housed either in SE or EE for 10 weeks. SE consisted in standard laboratory cages (20 × 35 × 16 cm), housing three animals per cage with food and water ad libitum. EE consisted in big cages (60 × 80 × 16 cm), housing six animals per cage and containing running wheels, ramps, tunnels, and different objects repositioned daily and fully substituted once a week (Fig. 1A). In some animals, exposure to EE was delayed (dEE) starting at 4 weeks post-SCGx (Fig. 3A). Animals were identified by earmarks, numbered ad hoc, and randomly assigned to SE or EE, or SE or dEE with a computer-based randomization method (https://www.graphpad.com/quickcalcs/randomize1).

### Visual stimulation

VS consisted in standard transparent laboratory cages housing three animals per cage surrounded by four PC monitors projecting 100% contrast black/white patterns for 6 s, followed by a 50% grey image for 12 s during the 12 h light phase [69] for 10 weeks. The control group (NVS) received a 50% grey image during the 12 h light phase (Fig. 4A). Animals were identified by earmarks, numbered ad hoc, and randomly assigned to NVS or VS with a computer-based randomization method (https://www.graphpad.com/quickcalcs/randomize1).

### ERG recording

Standard scotopic electroretinographic activity was assessed with a HMsERG model 2000 (Ocuscience LLC, Kansas City, MO, USA), as previously described [13, 14, 34]. A total of 12 eyes/group were averaged and the mean was taken as the representative value. 

### Behaviour visual tests

Looming, visual cliff and virtual visual cliff tests were used to evaluate visual functions in unilateral SCGx-submitted animals and unilateral sham-submitted animals as previously described [34]. A number of 12 mice/group were used for each test (only once for each mouse to avoid habituation).

### Electron microscopy

Ultrathin sections (50 nm) from the nasal and temporal retina (at 800 μm from the optic nerve head (ONH)) were obtained using glass knives and an ultramicrotome Ultracut E (Reichert-Jung, Vienna, Austria) and viewed and photographed using a Zeiss 109T transmission electron microscope (Carl Zeiss Microscopy, Peabody, MA, USA) equipped with a digital camera (ES1000W; Gatan, Pleasanton, CA, USA) as previously described [13, 14, 34]. Four different sections from 4 eyes/group were analysed.

### RPE melanin content quantification

Paraffin wax sections were deparaffinised dehydrated and mounted in Canada balsam without any other treatment and melanin content in the RPE of each sample at 800 μm of the ONH was quantified as previously described [13, 14, 34]. The average of four separate sections per eye, and the mean of five eyes were recorded as the representative value for each group.

### Immunofluorescence studies

Animals were intracardially perfused with saline, followed by 4% paraformaldehyde in PBS and paraffin sections were obtained and mounted in superfrost microscope slides (Erie Scientific Company, Portsmouth, New Hampshire, USA), as previously described [13, 14, 34]. Sections were preincubated with 5% normal horse serum for 1 h and then were incubated overnight at 4 °C with a mouse polyclonal anti-RPE65 antibody (1:500; EMD Millipore, Darmstadt, Germany, MAB5428), a mouse monoclonal anti-4HNE (1:250, R&D Systems, Minneapolis, MN, USA), or a mouse monoclonal anti-CML (1:250, R&D Systems, Minneapolis, MN, USA), a rabbit polyclonal anti-BDNF (1:250, Alomone Labs, Jerusalem, Israel), a mouse monoclonal anti-glutamine synthetase (GS) (1:500, Millipore, California, CA, USA). The following secondary antibodies were added and incubated for 2 h at room temperature: a goat anti-rabbit IgM secondary antibody conjugated to Alexa Fluor 488 (1:500; Invitrogen, Molecular Probes, Carlsbad, CA, USA, A11031) and a goat anti-mouse IgM secondary antibody conjugated to Alexa Fluor 568 (1:500; Invitrogen, Molecular Probes, Carlsbad, CA, USA, A11031). Nuclei were stained with Hoechst (1 μg/ml Sigma Chemical Co., St Louis, MO, USA), and observed under an epifluorescence microscope (BX50; Olympus, Tokyo, Japan) with a video camera attached to a computer running image analysis software (Image-Pro Plus, Media Cybernetics Inc., Bethesda, USA). The average of four separate sections per eye, and the mean of five eyes were recorded as the representative value for each group.

### Ex vivo flat-mounted RPE mitochondrial labelling and superoxide detection

RPE flat-mounts were obtained and incubated with 500 nM MitoTracker-Red CMXRos (Molecular Probes, Eugene, OR, USA) in MR buffer for 15 min or 5 µM MitoSOX-Red (Molecular Probes, Eugene, OR, USA) in MR buffer for 30 min at 37 °C in dim red light, as previously described [13, 14, 34]. For each group, four images from the central nasal and temporal RPE from five different eyes were analysed.

### Tissue harvesting for SDS-PAGE and western blotting

The neural retina was detached from the RPE, and both tissues were homogenized as previously described [13, 14, 34]. Proteins (50 μg/sample) were separated in SDS, 12% polyacrylamide gel, transferred to polyvinylidene difluoride membranes, and incubated overnight at 4 °C with a mouse monoclonal anti-cytochrome *c* (1:1000, Santa Cruz Biotechnology, Dallas TX, USA), a rabbit polyclonal anti-TOM20 (1:500, Santa Cruz Biotechnology, Dallas, TX, USA), a rabbit polyclonal anti-VDAC (1:300, Santa Cruz Biotechnology, Dallas, TX, USA), a rabbit polyclonal anti-BDNF (1:500, Alomone Labs, Jerusalem, Israel), a mouse polyclonal anti-RPE65 (1:500; EMD Millipore, Darmstadt, Germany, MAB5428) and a mouse anti-β-actin (1:1000, Santa Cruz Biotechnology, Dallas, TX, USA). The following secondary antibodies were used: a donkey anti-mouse (1:2000, Jackson Laboratory, Bar Harbor, ME, USA) and a donkey anti-rabbit (1:2000, Jackson Laboratory, Bar Harbor, ME, USA). Densitometric signals were quantified using ImageQuant software and adjusted by the density of β-actin. For each group, the mean of five homogenates were averaged and taken as the representative value.

## Statistical analysis

Experimenters were blinded to group assignment and outcome assessment for all experiments, and all involved observers. No sample size calculation was performed. Normality was assessed by Shapiro–Wilks test. Data are expressed as media ± SEM for two biological replicates, and comparison between groups was done by a two-way analysis of variance (ANOVA) followed by a Tukey’s test. The assumption of equal variances was tested by the *F*-test. In every statistical analysis, *P* < 0.05 was considered statistically significant.

## Supplementary information

Supplementary Figure Legends

Supplementary Table 1

Supplementary Figure 1

Supplementary Figure 2

Supplementary Figure 3

Supplementary Figure 4

Supplementary Figure 5

Supplementary Figure 6

## Data Availability

The datasets used and/or analysed during the current study are available from the corresponding author on reasonable request.

## Code availability

Heatmaps representing the mice tracked position on the virtual visual cliff arena (Figs. 1, 3 and 4); co-immunoreactivity heatmaps and average of the co-immunoreactivity pixel intensity bars between BDNF and GS, and BDNF and RPE65 (Supplementary Figs. 3 and 5); and distribution of green and red pixel intensity analysis along the retina (Supplementary Figs. 3 and 5) from BDNF, GS and RPE65 immunofluorescence images were done using python-based programming as stated at: https://github.com/hernanhd14/hernanhd14.git.
