# Supplementary material for: Enriched environment and visual stimuli protect the retinal pigment epithelium and photoreceptors in a mouse model of non-exudative age-related macular degeneration
Source: Cell Death Dis. 2021 Dec 4;12(12):1128. doi: 10.1038/s41419-021-04412-1 (PMC9632251; doi:10.1038/s41419-021-04412-1)
Supplement: Supplementary file 1 — Supplementary Figure Legends [file 41419_2021_4412_MOESM1_ESM.docx]

**SUPPLEMENTARY FIGURE LEGENDS**

**Supplementary Figure 1. Effect of EE on the choriocapillaris thickening induced by SCGx at 10 weeks post-surgery.** Panel A: SCGx induced a ubiquitous (i.e., the central nasal and the temporal region) significant increase in the choriocapillaris lumen height. EE did not prevent this alteration in SCGx-eyes (quantified in Panel B). Shown are representative photomicrographs from 4 animals/group, at 800 μm nasally and temporally from the ONH. Chr, choroid; Ch, choriocapillaris; Scale bar = 1 nm. Data are mean ± SEM (n: 4 animals per group), ***P* < 0.01 vs. sham-treated eyes from animals in SE, by Tukey´s test.

**Supplementary Figure 2. Effect of EE on the nasal outer retina/RPE structure at 10 weeks post SCGx.** Panel A: SCGx did not induce any alterations in BrM thickness and structure, RPE65-immunoreactivity, RPE melanin content, and RPE and PR integrity in eyes from animals housed in SE. EE had no effect both in sham- and SCGx-treated eyes (quantified in Panels B-D). Shown are representative photomicrographs from 5 (for optic microscopy) and 4 (for electronic microscopy) eyes/group, at 800 μm nasally from the ONH. OS, PR outer segments; RPE, retinal pigment epithelium; BI, RPE basal infoldings; BrM, Bruch´s membrane; Chr, choroid; Ch, choriocapillaris. Scale bars = 500 nm, 20 µm, 25 μm, 1.5 μm, 300 nm. Data are mean ± SEM (n: 4 animals per group (for electronic microscopy) and 5 animals per group (for optic microscopy)).

**Supplementary Figure 3. Effect of EE on the nasal outer retina/RPE oxidative stress markers, RPE mitochondria mass at 6 weeks post-SCGx.** Panels A and B: Neither SGCx nor EE had any effect in 4HNE- and CML-immunoreactivity at the nasal outer retina/RPE. Shown are representative photomicrographs at 800 μm nasally from the ONH from 5 eyes/group. ONL, outer nuclear layer; OS, PR outer segments; RPE, retinal pigment epithelium; Chr, choroid. Scale bars: 30 μm. Panel C: There were no differences in MitoSox-Red-labeled mitochondria or Mitotracker-Red(+) puncta between sham- and SCGx-treated eyes from animals housed in SE and EE. Shown are representative photomicrographs at 800 μm nasally from the ONH from 5 eyes/group. Scale bars = 20 μm. Data are mean ± SEM (n: 5 eyes per group). Panel D-G: No differences were found in the levels of cytochrome c, VDAC, and TOM20 at the nasal RPE from sham- and SCGx-treated eyes from animals in SE and EE. Data are mean ± SEM (n: 5 homogenates per group).

**Supplementary Figure 4. Effect of EE on the nasal retina/RPE BDNF-immunoreactivity and protein levels, and nasal retina BDNF/GS co-localization at 6 weeks post-SGCx.**

Panel A-C: There were no differences in BDNF-immunoreactivity and levels at the nasal retina and RPE between sham- and SCGx-treated eyes from SE and EE animals. Shown are representative photomicrographs at 800 μm nasally from the ONH from 5 eyes/group. RGC, retinal ganglion cell layer; IPL, inner plexiform layer; INL, inner nuclear layer; OPL, outer plexiform layer; ONL, outer nuclear layer; IS, PR inner segments; OS, PR outer segments; RPE, retinal pigment epithelium; Chr, choroid. Scale bars = 30 μm. Data are mean ± SEM (n: 5 homogenates per group). Panels D and E: No differences in BDNF/GS co-localization and the red and green pixel intensity were found between experimental groups. Shown are representative filtered co-immunoreactivity python-based analysis, average of the co-immunoreactivity pixel intensity bars, and python-based distribution of green pixel (BDNF) and red pixel (GS) intensity analysis along the retina from representative retinal photomicrographs at 800 μm nasally from the ONH from 5 eyes/group. RGC, retinal ganglion cell layer; IPL, inner plexiform layer; INL, inner nuclear layer; OPL, outer plexiform layer; ONL, outer nuclear layer; IS, PR inner segments. Scale bar = 30 μm.

**Supplementary Figure 5. Effect of dEE on the nasal RPE structure at 10 weeks post-SCGx.** Panel A: There were no differences in RPE melanin content and RPE65-immunoreactivity between experimental groups (quantified in Panels B and C). Shown are representative photomicrographs at 800 μm nasally from the ONH from 5 eyes/group. OS, PR outer segments; RPE, retinal pigment epithelium; Chr, choroid. Scale bars = 25 μm. Data are mean ± SEM (n: 5 eyes per group).

**Supplementary Figure 6. Effect of VS on the nasal RPE structure at 10 weeks post-SCGx.** Panel A: There were no differences in RPE melanin content and RPE65-immunoreactivity between experimental groups (quantified in Panels B and C). Shown are representative photomicrographs at 800 μm nasally from the ONH from 5 eyes/group. OS, PR outer segments; RPE, retinal pigment epithelium; Chr, choroid. Scale bars = 25 μm. Data are mean ± SEM (n: 5 eyes per group).
