# Supplementary material for: Enriched environment and visual stimuli protect the retinal pigment epithelium and photoreceptors in a mouse model of non-exudative age-related macular degeneration
Source: Cell Death Dis. 2021 Dec 4;12(12):1128. doi: 10.1038/s41419-021-04412-1 (PMC9632251; doi:10.1038/s41419-021-04412-1)
Supplement: Supplementary file 2 — Supplementary Table 1 [file 41419_2021_4412_MOESM2_ESM.docx]

**Supplementary Table 1. Effect of EE, dEE and VS on ERG b-wave amplitude, and ERG a- and b-wave latencies at 10 weeks post-SCGx.**

|  | ERG b-wave amplitude (μV) | ERG a-wave latency (msec) | ERG b-wave latency (msec) |
| --- | --- | --- | --- |
| Sham + SE | 405.5 ± 50.5 | 20.5 ± 1.2 | 44.4 ± 2.8 |
| Sham + EE | 467.5 ± 46.7 | 21.3 ± 1.5 | 40.8 ± 2.7 |
| SCGx + SE | 489.2 ± 41.1 | 19.1 ± 1.7 | 42.3 ± 2.2 |
| SCGx + EE | 510.3 ± 59.9 | 19.8 ± 1.3 | 46 ± 3.1 |
| Sham + SE | 428.4 ± 65.5 | 18.9 ± 1.8 | 41.5 ± 2.4 |
| Sham + dEE | 435.9 ± 36.7 | 19.7 ± 2.2 | 44.3 ± 3.2 |
| SCGx + SE | 473.2 ± 44.9 | 21.2 ± 2.5 | 45.1 ± 2.7 |
| SCGx + dEE | 490 ± 45.5 | 20.5 ± 1.9 | 42.9 ± 1.9 |
| Sham + NVS | 448.1 ± 75.6 | 23.5 ± 2.8 | 44.7 ± 2.1 |
| Sham + VS | 479.5 ± 41.3 | 22.5 ± 1.9 | 41.3 ± 2.5 |
| SCGx + NVS | 483.6 ± 64.7 | 23.8 ± 2.2 | 42.5 ± 2.8 |
| SCGx + VS | 469.6 ± 81.9 | 20.2 ± 1.7 | 43.5 ± 1.9 |

n=12 eyes per group, ns by Tukey´s test.
