# Supplementary figures and images for: Enriched environment and visual stimuli protect the retinal pigment epithelium and photoreceptors in a mouse model of non-exudative age-related macular degeneration
Source: Cell Death Dis. 2021 Dec 4;12(12):1128. doi: 10.1038/s41419-021-04412-1 (PMC9632251; doi:10.1038/s41419-021-04412-1)

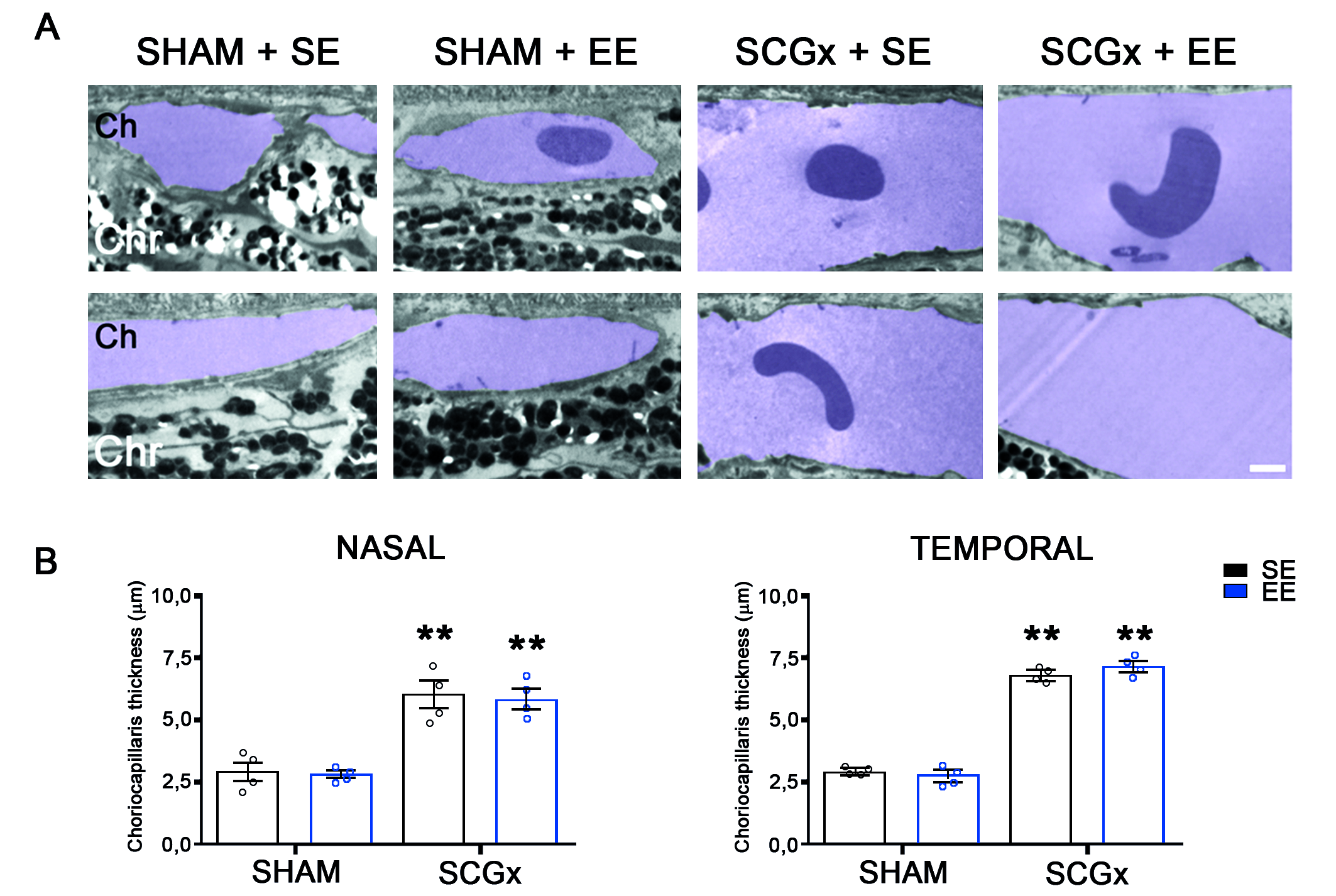

Supplement: Supplementary file 3 — Supplementary Figure 1 [file 41419_2021_4412_MOESM3_ESM.tif]

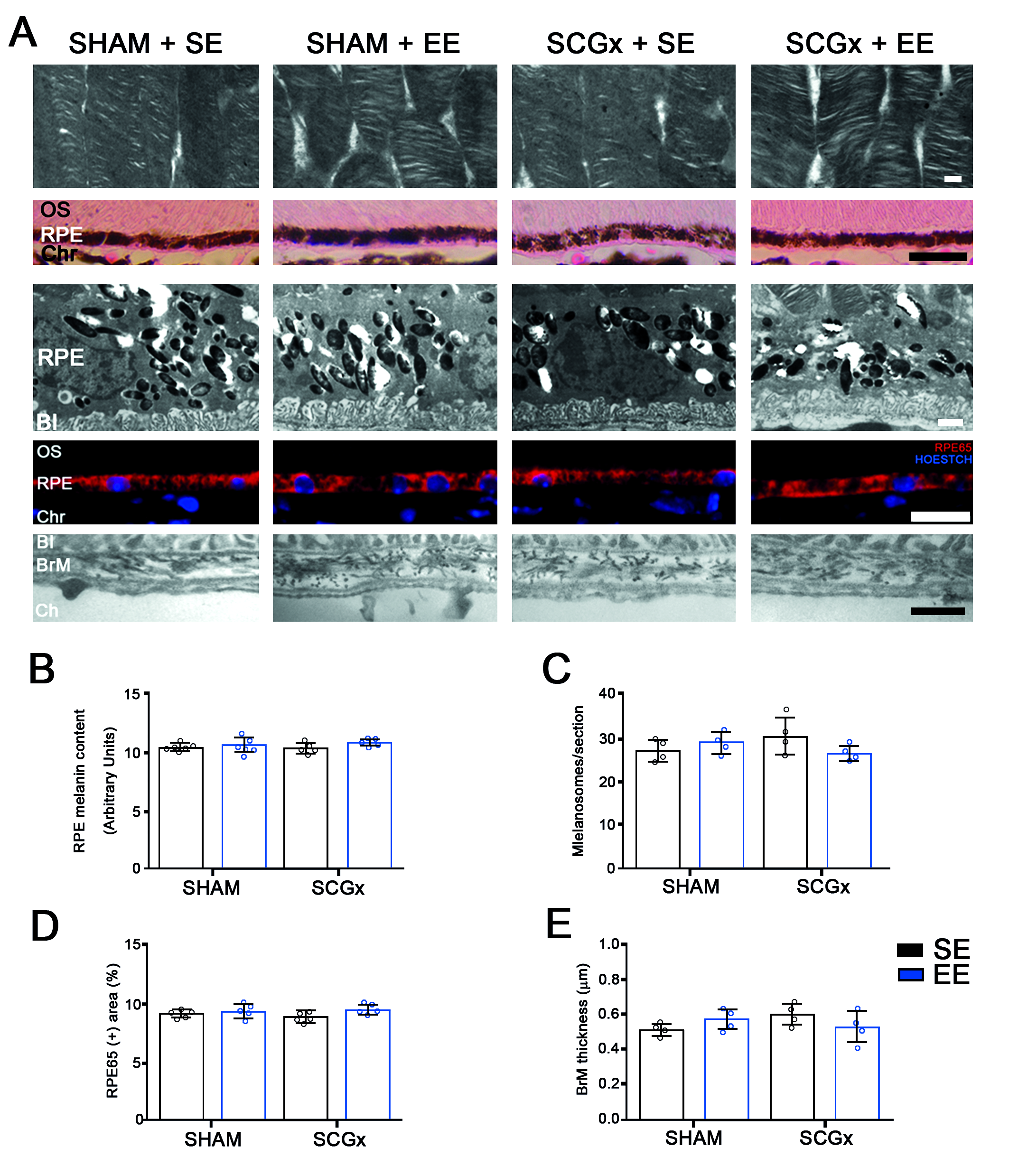

Supplement: Supplementary file 4 — Supplementary Figure 2 [file 41419_2021_4412_MOESM4_ESM.tif]

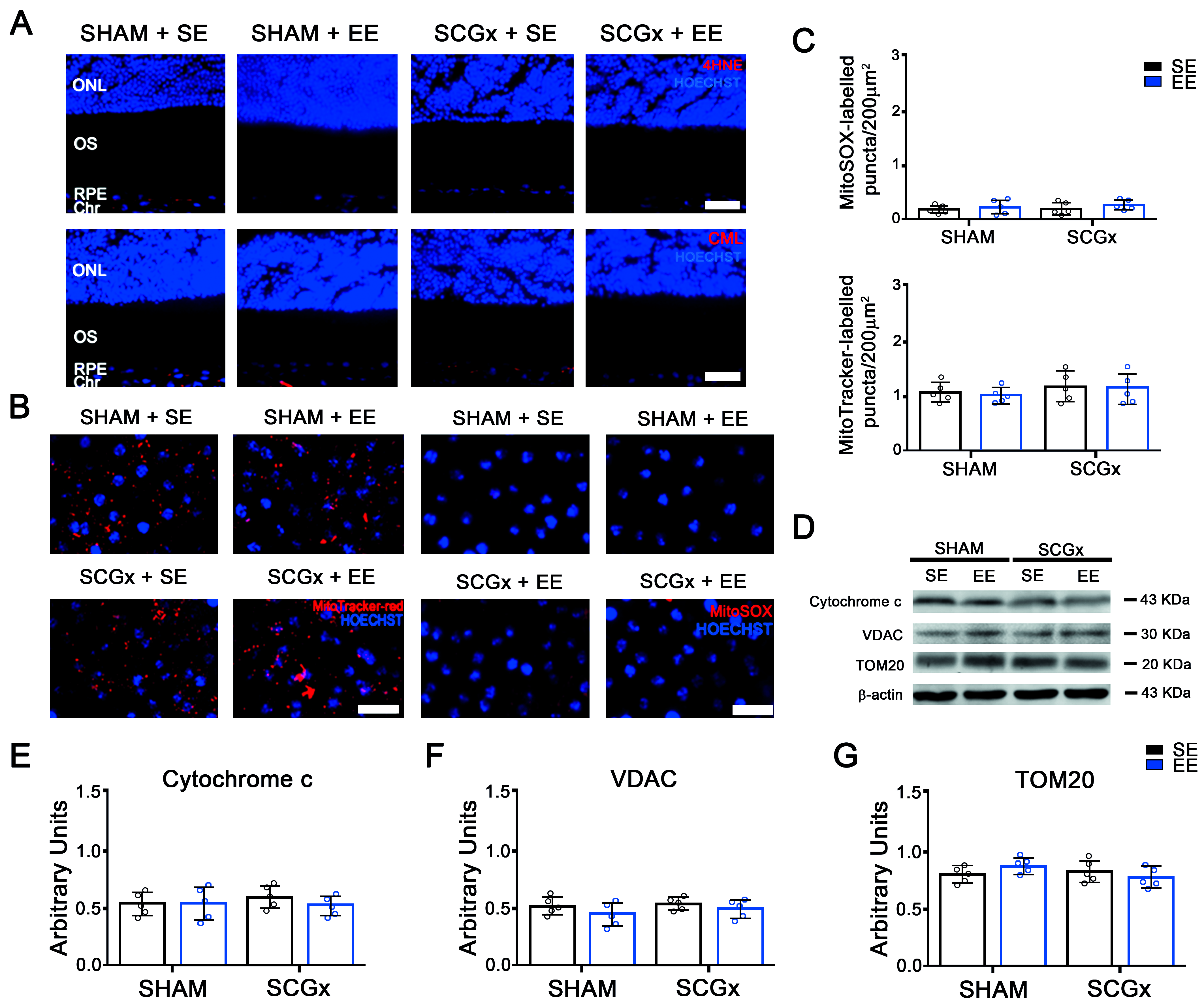

Supplement: Supplementary file 5 — Supplementary Figure 3 [file 41419_2021_4412_MOESM5_ESM.tif]

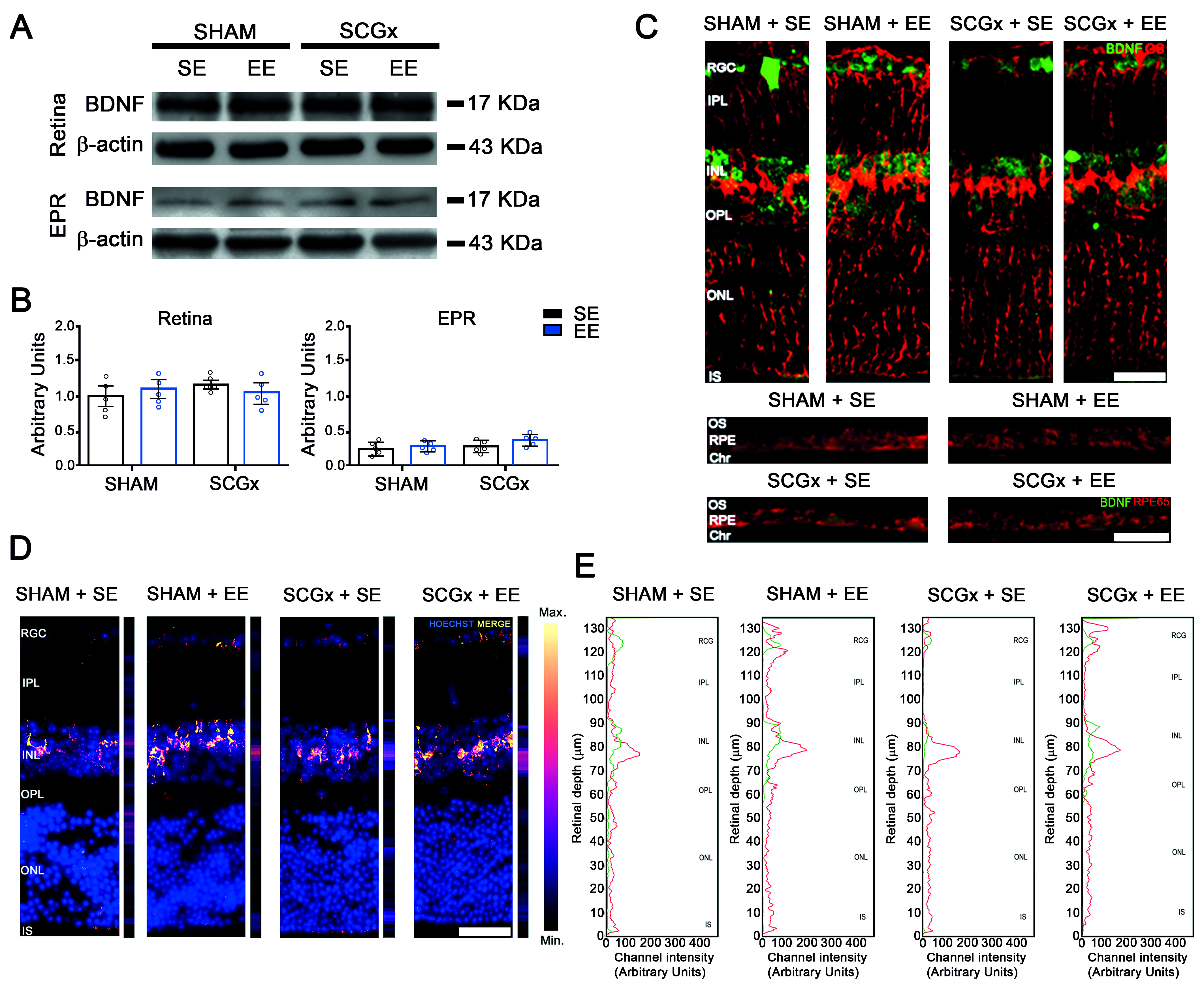

Supplement: Supplementary file 6 — Supplementary Figure 4 [file 41419_2021_4412_MOESM6_ESM.tif]

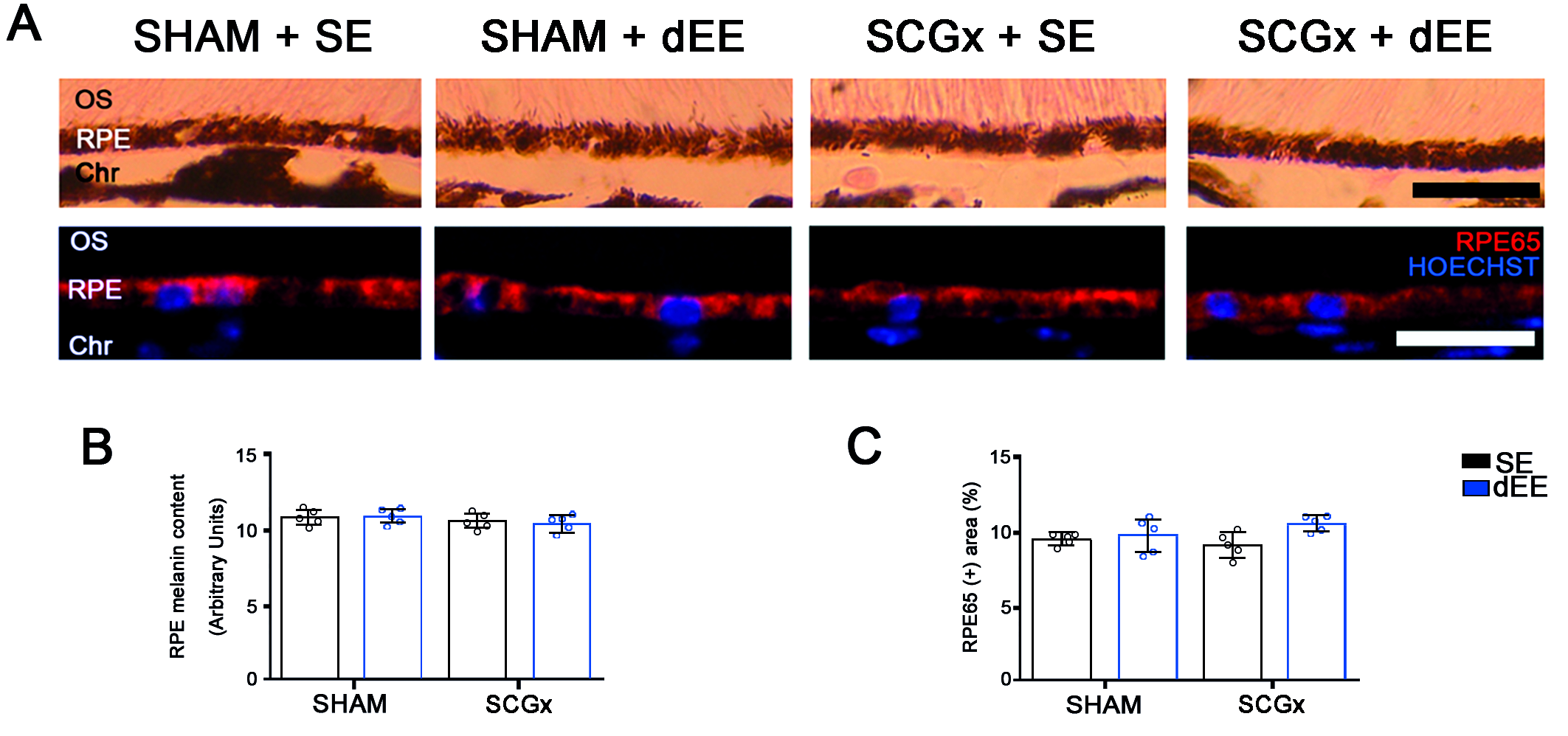

Supplement: Supplementary file 7 — Supplementary Figure 5 [file 41419_2021_4412_MOESM7_ESM.tif]

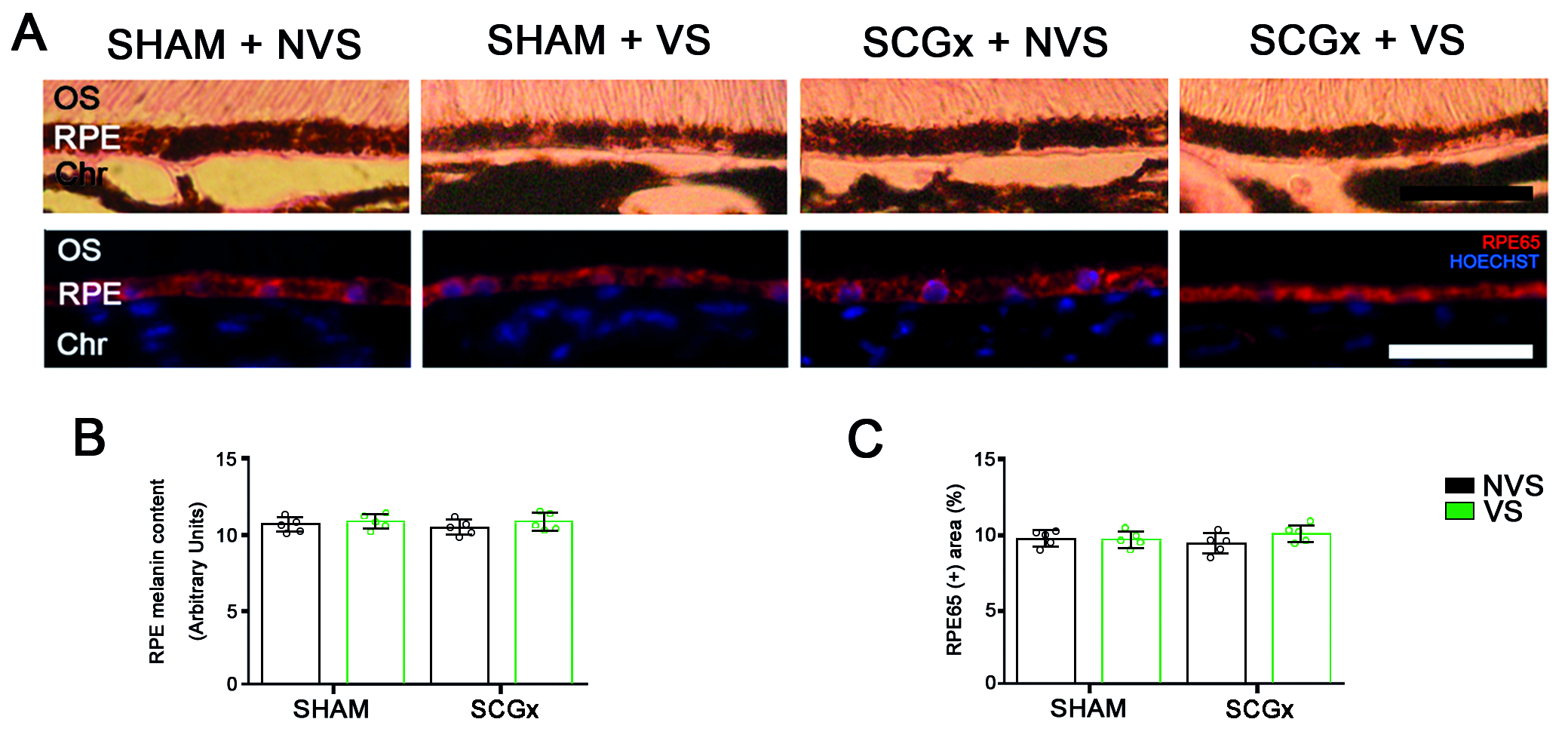

Supplement: Supplementary file 8 — Supplementary Figure 6 [file 41419_2021_4412_MOESM8_ESM.tif]
